# Continuous glucose monitoring reveals similar glycemic variability in individuals with obesity despite increased HOMA-IR

**DOI:** 10.3389/fnut.2022.1070187

**Published:** 2022-12-02

**Authors:** Dylan J. Cooper, Sharon Zarabi, Brianna Farrand, Amanda Becker, Mitchell Roslin

**Affiliations:** ^1^Department of Surgery, Northwell Health-Lenox Hill Hospital, New York, NY, United States; ^2^Donald and Barbara Zucker School of Medicine at Hofstra/Northwell Health, Hempstead, NY, United States; ^3^Northern Westchester Hospital, Mount Kisco, NY, United States

**Keywords:** continuous glucose monitoring, glycemic variability, insulin resistance, obesity, obesity management, HOMA-IR

## Abstract

**Background/aims:**

Continuous glucose monitoring is a well-tolerated and versatile tool for management of diabetes and metabolic disease. While its use appears to be feasible to monitor glycemic profiles in diabetics, there is a paucity of data in individuals with obesity and normal glucose tolerance. The aim of this study is to investigate glucose fluctuations and insulin resistance patterns in normoglycemic participants with obesity vs. without obesity and contextualize these results against leading models for obesity.

**Materials and methods:**

We designed a prospective, observational pilot study of two cohorts including 14 normoglycemic participants with obesity and 14 normoglycemic participants without obesity. Participants were monitored with continuous glucose monitoring (CGM) for five consecutive days. Insulin resistance levels were measured and glucometric data were extracted from CGM for all participants.

**Results:**

Fasting serum insulin and homeostasis model assessment of insulin resistance (HOMA-IR) were significantly higher in the group with obesity (*P* < 0.05). While the group with obesity had a higher mean blood glucose (MBG), mean amplitude of glycemic excursions (MAGE), and continuous overall glycemic action-1 h (CONGA-1), these differences were not significant. On univariate linear regression, insulin resistance (HOMA-IR) was associated with body mass index (BMI), waist circumference (WC), cohort with obesity, cohort consuming a high glycemic diet, hemoglobin A1c (HbA1c), and fasting insulin levels. WC and fasting insulin levels remained predictors of HOMA-IR in our multivariable model.

**Conclusion:**

While there is much excitement surrounding the use of commercial CGM products in obesity management, our results suggest that fasting insulin and HOMA-IR values may be more clinically useful than CGM data alone.

## Introduction

The obesity epidemic is a significant public health challenge as the proportion of American adults who are overweight or obese continues to increase (1). The exact etiology of the obesity epidemic remains a matter of debate. An improved understanding and identification of key triggers could result in better prevention and treatment strategies.

Fundamentally, obesity corresponds to excess adiposity. As lipids are anhydrous, they are an efficient means of storing excess energy in a relatively small area. At its core, obesity is the result of excess energy storage and reduced fat oxidation for cellular energy use. This storage process is tightly monitored by the central nervous system, regulated by the hypothalamus, the most important region involved in energy homeostasis (2, 3). Studies have shown either ablation or stimulation of different areas within the hypothalamus can result in hyperphagia or hypophagia due to disruption of the hypothalamic-adipose axis (4–6).

The idea of caloric intake exceeding energy expenditure represents the Energy Balance model for obesity, whereby the brain acts like a thermostat receiving input from numerous sensory pathways and reflexively up-regulates or down-regulates energy intake and expenditure to achieve homeostasis (7, 8).

While weight loss programs have targeted the century-old concept of reducing total caloric intake and increasing expenditure, weight management is not as simple as balancing a checkbook. When intake declines, the body responds by becoming more efficient and utilizing fewer calories; when activity is increased, appetite is increased to consume a greater amount of food (9, 10).

A second model for obesity, the Carbohydrate-Insulin model, posits that a high-carbohydrate diet drives post-prandial hyperinsulinemia, leading to increased fat storage. This occurs instead of oxidation by metabolically active tissues and pre-disposes to weight gain through increased hunger and a slowed metabolic rate (7, 8). Stated simply, this model prioritizes *what* is eaten, not simply total caloric value, in precipitating hyperinsulinemia.

Increased consumption of carbohydrates with high glycemic indices increases insulin secretion. The increased insulin drives nutrients into fat cells, leaving fewer nutrients for other tissues and stimulating increased food intake. Rather than a central model, the development of obesity occurs through peripheral mechanisms (11).

In line with this theory, modern diets including increased processed, high glycemic-load foods cause hormonal changes that lead to insulin production and promote adiposity (12). Animal studies have shown dietary composition is demonstrated to affect metabolism independent of caloric intake. Rodents fed high vs. low glycemic load diets controlled for macronutrients (carbohydrate, fat, and protein) produce a sequential series of endocrine dysfunction involving hyperinsulinemia, increased adipocyte diameter and other anabolic changes, including greater adiposity, lower energy expenditure, and increased hunger (7, 13–15).

Continuous Glucose Monitoring (CGM), which measures users’ glucose concentrations in the interstitial fluid, has made a profound impact on the management of diabetes (16, 17). CGM has been shown to improve quality of glycemic control and quality of life by avoiding multiple finger-sticks, reduce risk of hypoglycemia, obtain far more accurate readings, and enable lower target levels for mean glucose and hemoglobin A1c (HbA1c) for patients with diabetes (18–21).

CGM technology is increasingly cost-effective as factory-calibrated, disposable monitors are now publicly available (22). As a result, many have hypothesized that CGM could function as a dietary Fitbit™ and potentially alter behavior and intake of high glycemic foods.

For advocates of the Carbohydrate Insulin model, the argument is simple: as glucose spikes drive insulin, reducing glucose levels will lower insulin secretion and result in better weight control. Thus preventing glucose spikes and using real-time glycemic biofeedback may offer a more optimized, personalized, and effective weight loss program.

While the thought process is compelling, there is currently little evidence to support CGM usage in individuals with obesity and normal glucose tolerance. This study was undertaken to investigate the clinical utility of CGM—using indicators such as mean amplitude of glycemic excursions (MAGE), standard deviation of blood glucose (SDBG), mean of daily differences (MODD), and continuous overlapping net glycemic action over 1 h (CONGA-1) as indices for glycemic variability—in normoglycemic adults with obesity (BMI > 30) as compared to a group of normoglycemic adults without obesity (BMI < 30).

Using CGM data from these two groups may offer insight into the plausibility of the Energy Balance model for obesity versus the Carbohydrate-Insulin model. If the Carbohydrate-Insulin model best reflects the etiology of obesity, individuals with obesity should have increased glucose variability with higher amplitude glucose spikes and lower nadirs. But if rising insulin resistance is a consequence of excess adiposity, nutrient overload, and caloric imbalance, we would expect there to be a difference in fasting insulin and insulin resistance, but little difference in glucose variability. Rising insulin would be a compensatory response to regulate glucose. Further, if there is no significant difference in glycemic variability between the two groups, the use of CGM for the treatment of obesity may be less clinically beneficial in this study population than those marketing CGM technology have suggested.

We present a pilot study of 28 participants in which we compare (i) MAGE as a proxy for glycemic variability and (ii) levels of insulin, insulin resistance, and glucometric data extracted from CGM in a cohort of adults with obesity vs. without obesity.

## Materials and methods

### Study design

We performed a prospective, single institution, observational study from June 2020 to May 2021 of 14 adults with obesity (BMI > 30) and 14 adults without obesity (BMI < 30) who were all between 18 and 50 years of age. Participants were healthy adults, primarily recruited from family, neighbors, and patients seen in the bariatric surgery clinic. The inclusion criteria consisted of the following: (i) participants were clinically stable with no known chronic illness that might affect glucose metabolism including history of hypertension, dyslipidemia, coronary artery disease, or cerebral stroke; (ii) participants had point-of-care HbA1c values <5.7% (39 mmol/mol). Exclusion criteria included (i) previous history of bariatric surgery, and use of antihypertensive, anti-diabetic, thiazide diuretic, or cholesterol-lowering medications; (ii) female participants pregnant at the time of study enrollment; (iii) participants with known hepatic or renal dysfunction. The study was conducted within the Northwell Health Department of Bariatric Surgery after approval by institutional review board. Study participants provided written informed consent prior to study participation.

### Anthropometric measurements

Anthropometric measurements, including height, weight, and waist circumference (WC) were obtained as patients were enrolled. BMI was calculated by dividing weight (kg) by height squared (m^2^). The average BMI in the group with obesity was 38.4 kg/m^2^ and the average BMI in the group without obesity was 23.7 kg/m^2^.

### Laboratory examinations

Prior to implantation, fasting glucose and fasting insulin were measured to render a HOMA-IR score for each participant. HOMA-IR scores were calculated by [fasting glucose (mg/dl) × fasting insulin (μU/ml)]/405. HbA1c values were also measured.

### Continuous glucose monitoring

**FIGURE 1:**
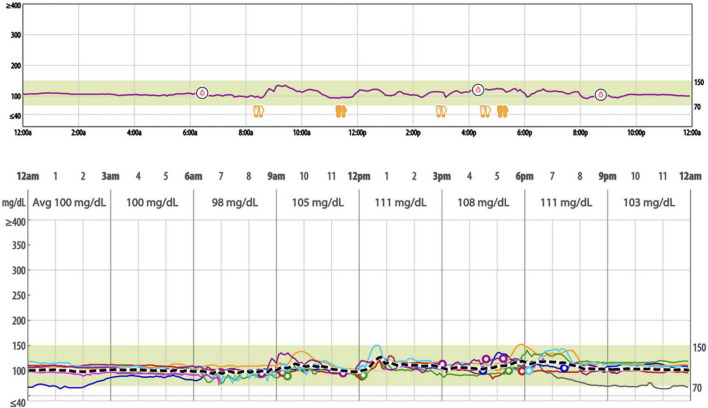
Example of a 24-hour period of continuous glucose monitoring with iPro2 and overlayed weeklong sensor data (mg/dl).

All participants were equipped with *i*Pro2 continuous glucose recorder (Medtronic, Northridge, CA, USA). On day 0, a CGM sensor (Enlite Sensor) was inserted into the subcutaneous abdominal fat tissue and calibrated according to standard Medtronic operating guidelines. The *i*Pro2 continuous glucose recorder measures subcutaneous tissue interstitial glucose levels continuously, recording values every 5 min, within a range of 40–400 mg/dl. With the *i*Pro2 CGM inserted, patients checked their blood glucose levels three times daily with a OneTouch^®^ Verio™ Flex meter (LifeScan, Malvern, PA, USA) to calibrate the *i*Pro2 continuous glucose sensor. On day 5, the participants returned to the research site, and the monitors were removed. The recorded data, including range, SD, glycemic variability indexes, and mean blood glucose were downloaded with Medtronic’s CareLink System and stored in a secure Northwell REDCap database for further analysis. Figure 1 shows an example of aggregate data from a 24-h period of CGM.

### Diet assessments

Participants were asked to keep detailed food logs by entering all foods consumed during the study period into the *i*Pro2 myLog cellphone app, as well as capture corresponding photos of all foods consumed. Participants followed an *ad libitum* diet. Total net carbs were estimated by two Registered Dietitians for each participant, and diets were then classified as being high or low in total net carbs. Carbohydrate information for each meal submission was obtained from the Carb Manager app (Wombat Apps LLC, Redmond, WA, USA). Diets classified as “high” contained the highest proportion of carbohydrate intake from refined sources. Diets classified as “low” included the highest proportion of fiber from vegetables and whole foods. Post-prandial peaks for each meal were recorded and average peaks for each participant with obesity vs. without obesity were analyzed to determine if meals of certain glycemic load were associated with blood sugar peaks.

### Assessment of glycemic variability

Glycemic variability was calculated using EasyGV version 9.0.R2 (© University of Oxford). The metrics generated from EasyGV in this analysis are mean of daily differences (MODD), mean amplitude of glycemic excursion (MAGE), continuous overlapping net glycemic action over 1 h (CONGA1), and the standard deviation of the glucose values (SDBG).

### Statistical analyses

All analyses were performed using the SPSS 16.0 statistical software for Windows (SPSS, Chicago, IL, USA). Values are shown as mean with standard deviation. Fasting glucose, fasting insulin, HbA1c, and HOMA-IR scores were imported into the REDCap database. Calculations for range of glucose values and MAGE from the CGM data were performed using the nadir-to-peak excursions. The two groups were compared using the Student *t*-test. A univariate linear regression analysis was performed to explore predictors of both glycemic variability (MAGE) and insulin resistance (HOMA-IR) for the following set of covariates: age, female gender, member of adult with obesity cohort, high glycemic load diet during study, fasting plasma glucose and serum insulin, and specific CGM metrics. Parameters statistically significant on univariate analysis were considered for a multivariable linear regression analysis. *P* < 0.05 was considered statistically significant.

## Results

We enrolled 30 participants for this pilot study: two were excluded from analysis due to missing CGM values. Data were analyzed from 28 participants, ranging from 21 to 49 years, 14 of whom were adults with obesity (BMI > 30 kg/m^2^) and 14 were adults without obesity (BMI < 30 kg/m^2^). The baseline characteristics for the two cohorts were similar with respect to age, gender, and race/ethnicity, as shown in Table 1. BMI, WC, fasting serum insulin, and HOMA-IR were all significantly higher in the group with obesity than in the group without obesity (*P* < 0.05). No significant differences between the two groups were observed in HbA1c and fasting plasma glucose levels, confirming normoglycemia.

**TABLE 1:** Baseline demographic and clinical characteristics of the participants in each group.

	Group without obesity	Group with obesity	Statistics
Age (years) mean	30.1 ± 7.49	32.4 ± 8.54	*t* = 0.753, *p* = 0.458
Gender Male	4 (28.6%)	5 (35.7%)	χ*^2^* = 0.164, *p* = 0.686
Race/Ethnicity Caucasian Black Hispanic Other	5 (35.7%) 2 (14.3%) 3 (21.4%) 4 (28.6%)	6 (42.9%) 3 (21.4%) 2 (14.3%) 3 (21.4%)	χ*^2^* = 0.634, *p* = 0.889
BMI (kg/m^2^) mean	23.7 ± 2.2	38.4 ± 5.9	*t* = 8.73, *p* = 0.000*
Waist circumference (cm) mean Male Female	87.0 ± 1.27 78.4 ± 5.12	108.4 ± 5.90 95.0 ± 6.50	*t* = 13.27, *p* = 0.000* *t* = 7.51, *p* = 0.000*
HbA1c mean	5.27 ± 0.31	5.39 ± 0.22	*t* = 1.18, *p* = 0.248
Fasting plasma glucose (mg/dl) mean	85.4 ± 6.69	89.1 ± 4.40	*t* = 1.17, *p* = 0.096
Fasting serum insulin (μU/ml) mean	6.19 ± 2.12	17.32 ± 7.61	*t* = 5.27, *p* = 0.000*
HOMA-IR mean	1.30 ± 0.47	3.8 ± 1.64	*t* = 5.48, *p* = 0.000*

Statistics are presented using *t*-test for the continuous variables (all denoted by means with standard deviations) and a chi-square test for categorical variables.

*Significant difference between the two groups.

All participants included in the study successfully calibrated their CGM monitors on the day of implantation. Once calibrated, CGM data were generated for an average of 5.09 days [range 4–6]. Table 2 shows the data extracted from CGM. While the group with obesity had a higher mean blood glucose (MBG), mean amplitude of glycemic excursions (MAGE), and continuous overall glycemic action-1 h (CONGA-1) than the group without obesity, these differences were not found to be significant. The standard deviation of blood glucose (SDBG) and mean of daily differences (MODD) were found to be higher, although not significantly, in the group without obesity than in the group with obesity.

**TABLE 2:** Results extracted from continuous glucose monitoring and dietary logs in each group.

	Group without obesity	Group with obesity	Statistics
Mean blood glucose (mmol/l)	5.50 ± 0.39	5.69 ± 0.35	*t* = 1.36, *p* = 0.187
Mean amplitude of glycemic excursions (mmol/l)	1.26 ± 0.56	1.46 ± 0.37	t = 1.11, *p* = 0.275
Standard deviation of blood glucose (mmol/l)	0.92 ± 0.32	0.80 ± 0.18	*t* = 1.22, *p* = 0.232
Continuous overall net glycemic action-1 h (mmol/l)	5.11 ± 0.40	5.34 ± 0.27	*t* = 1.78, *p* = 0.090
Mean of daily differences (mmol/l)	0.91 ± 0.25	0.80 ± 0.13	*t* = 1.46, *p* = 0.156
% High glycemic load diet	18.18%	66.67%	χ*^2^* = 4.85, *p* = 0.028*

Statistics are presented using *t*-test for the continuous variables (all denoted by means with standard deviations) and a chi-square test for categorical variables.

*Significant difference between the two groups.

For the diet assessment, only 20 of the 28 participants maintained detailed food logs with corresponding photo submissions. Based on the limited data, two Registered Dietitians focused the analysis on carbohydrate quality and quantity—estimating the proportion of refined carbohydrate versus fiber, as well as the total carbohydrate content of the meal. Of the 20 diets that could be analyzed, 12 were low glycemic load and 8 were high glycemic load. Chi-square analysis showed that of the 20 adults who kept dietary logs, there was a difference in glycemic load between the two cohorts, as seen in Table 2.

Tables 3 and 4 show the covariates analyzed through linear regression analysis. When assessing glycemic variability and insulin resistance (using MAGE and HOMA-IR as proxies respectively), only HOMA-IR demonstrated significant results. BMI, WC, adults with obesity, high glycemic diet, HbA1c, and fasting insulin levels maintained an independent association with HOMA-IR. The multivariable model was significant [*F*(6,19) = 367.80, *p* < 0.001] accounting for 99% of the variance (adjusted *R*^2^). WC [β = 0.019 (0.005–0.033), *p* = 0.010] and fasting insulin levels [β = 0.212 (0.193–0.232), *p* < 0.001] were found to be predictors of HOMA-IR.

**TABLE 3:** Univariate linear regression analysis of factors associated with mean amplitude of glycemic excursions (MAGE).

	Univariate analysis	
Covariate	β *[95% CI]*	*P-*value
Age (years)	0.005 [−0.019–0.029]	0.672
Gender Male Female	Ref 0.049 [−0.352–0.450]	0.803
BMI (kg/m^2^)	0.011 [–0.011–0.032]	0.310
WC (cm)	0.009 [–0.006–0.025]	0.231
Adult with obesity cohort	0.198 [–0.168–0.565]	0.275
High glycemic load diet	0.310 [–0.144–0.765]	0.169
Fasting plasma glucose (mmol/l)	0.019 [–0.013–0.050]	0.231
HbA1c	0.268 [–0.414–0.950]	0.427
Fasting insulin (μU/ml)	0.010 [–0.014–0.034]	0.863
HOMA-IR	0.046 [–0.061–0.153]	0.880
Standard deviation of blood glucose (mmol/l)	0.047 [–0.686–0.781]	0.895
Continuous overall net glycemic action-1 h (mmol/l)	0.404 [–0.108–0.916]	0.117
Mean of daily differences (mmol/l)	–0.004 [–0.919–0.910]	0.992

**TABLE 4:** Univariate and multivariable linear regression analysis of factors associated with HOMA-IR.

Covariate	Univariate analysis		Multivariable analysis	
	β *[95% CI]*	*P-*value	β *[95% CI]*	*P*-value
Age (years)	−0.019 [−0.108–0.069]	0.660		
Gender Male Female	Ref −0.773 [−0.227–0.682]	0.285		
BMI (kg/m^2^)	0.155 [0.103–0.207]	0.000*	0.004 [−0.017–0.025]	0.666
WC (cm)	0.107 [0.065–0.148]	0.000*	0.019 [0.005–0.033]	0.010*
Adult in obesity cohort	2.493 [1.534–3.452]	0.000*	−0.276 [−0.616–0.064]	0.103
High glycemic load diet	0.092 [−0.023–0.206]	0.000*	0.026 [−0.199–0.250]	0.808
Fasting plasma glucose (mmol/l)	−0.083 [−0.180–0.014]	0.112		
HbA1c	2.737 [0.427–5.047]	0.022*	−0.051 [−0.404–0.301]	0.758
Fasting insulin (μU/ml)	0.222 [0.214–0.230]	0.000*	0.212 [0.193–0.232]	0.000*
MAGE	0.046 [−0.061–0.153]	0.880		
Standard deviation of blood glucose (mmol/l)	−1.542 [−4.188–1.1.05]	0.242		
Continuous overall net glycemic action-1 h (mmol/l)	1.274 [−0.650–3.198]	0.185		
Mean of daily differences (mmol/l)	−2.222 [−5.492–1.048]	0.174		

*Denotes statistical significance.

## Discussion

This pilot study, which recruited normoglycemic men and women of diverse backgrounds, showed that glucometric data measuring glucose variability was similar in groups with obesity and without obesity. In contrast, even in this small sample, fasting insulin and HOMA-IR were significantly higher in participants with obesity. Surprisingly, while not statistically significant, the standard deviation of blood glucose was higher in participants without obesity.

On linear regression analysis, we found associations with HOMA-IR—and not MAGE—for BMI, WC, HbA1c, and fasting insulin levels. On multivariable analysis, WC and fasting insulin levels remained significantly associated with HOMA-IR. Taken together, these preliminary results suggest that the rise in insulin may be secondary to the development of insulin resistance and a compensatory mechanism in the glucose regulation process.

The results of this study lend support to the Energy Balance model for obesity. If a high glycemic load were the impetus for development of obesity, then greater glycemic variability would be seen in our cohort with obesity, which was not the case. To obtain further insight, we had patients keep dietary logs that were reviewed by Registered Dietitians, which showed that although our cohort with obesity on average ate meals with a higher glycemic load, their glycemic variability was not different from that of the cohort without obesity. However, we acknowledge that our small sample size and the difficulty in obtaining accurate dietary logs limits interpretation of these results.

Consistent with this are studies that demonstrate that excess nutrients within the skeletal muscle cells signal the cell membrane to block insulin-dependent glucose uptake by muscle cells. Petersen KF et al. demonstrated that insulin-resistant individuals have marked defects in muscle glycogen synthesis and divert their ingested energy into hepatic *de novo* lipogenesis. When insulin-resistant individuals are challenged with a high glycemic meal challenge, their post-prandial plasma glucose concentrations were similar to insulin-sensitive individuals (23).

For CGM to be effective, it must be assumed that glucose values are reflective of insulin secretion. However, this assumption has not been proven to be true in normoglycemic populations. Furthermore, excess fructose has been implicated in the development of obesity, diabetes, cardiovascular disease, non-alcoholic fatty liver disease, and cancer (24–28). Fructokinase C, present in the liver, converts fructose into metabolites such as citrate and uric acid that result in the net breakdown of ATP and endothelial dysfunction (29). Interestingly, fructose ingestion does not markedly raise glucose values as both dextrose or sucrose do and thus its intake would not be sharply detected by CGM (30, 31).

The data for the use of CGM devices in normoglycemic individuals with obesity are not robust (32). Salkind et al. (33) performed an observational study using CGM that compared contestants on the Biggest Loser reality show who were morbidly obese and were either normoglycemic or pre-diabetic. While there was no difference in glycemic variability between the two groups, Salkind et al. state that both groups had greater glycemic variability as compared with historical controls. However, one limitation of this study, and many other CGM studies in the literature, is that there is no contemporaneously studied normal weight control group, which our study indeed has (34).

In another study, Ma et al. (35) demonstrate that MBG levels and glycemic variability were increased in abdominally obese men with normal glucose tolerance who were of Han ethnicity. In contrast to our study, there was a difference in baseline mean blood glucose within their two cohorts, a smaller difference in BMI, and they only analyzed males with central obesity. Within Asian populations, the prevalence of diabetes with lower body mass index levels is well-documented (36), and we speculate that many of the patients in this cohort may have been pre-diabetic as baseline HbA1c was not documented.

More consistent with our findings is a recent study using CGM in adolescents with obesity (37). Investigators used CGM to compare whether glucose variability is altered during time-restricted eating (TRE). They found no difference in variability when TRE was compared to a diet that was not time limited. Theoretically, time restriction lowers insulin levels. The absence of any difference could be that TRE as used in this study does not result in the anticipated reduction in insulin levels, or that glucose value is not a sensitive method of measuring insulin.

While it is known that dietary interventions for normalizing glycemic levels are associated with changes in health markers, including fasting blood glucose level and HbA1c, the role of CGM in weight management is not established (38, 39). Although the idea of CGM translating to improved health outcomes is compelling, the commercialization of this technology has begun without clinical, peer-reviewed evidence of efficacy for weight loss.

Advocates of CGM have suggested that nutrition can be personalized by identifying specific foods that cause spikes in glucose levels. However, the standard American diet involves consumption of a variety of food groups at the same time and furthermore, failure to raise blood sugar levels is not synonymous with healthy eating. When a sugary dessert is eaten after a heavy meal, it causes less of a rise in blood sugar than when eaten on an empty stomach (40). The temporal sequence of carbohydrate ingestion during a meal has a significant impact on post-prandial glucose excursions.

Thus, there are many factors that contribute to glucose excursions or the lack thereof; and while CGM can identify glucose excursions, its role in weight loss for the modern consumer is not established. Our pilot study does not eliminate the possibility that if a participant wore a glucose monitor and tailored one’s diet to lower glucose levels, that there would be weight loss. However, our results point toward metrics for insulin resistance, such as HOMA-IR, as potentially stronger clinical markers for dysglycemia and metabolic syndrome.

Furthermore, there may be harm related to the use of CGM in its capacity to raise false alarms and lead to unnecessary healthcare use, including excess clinical visits and inappropriate medication administration. Users may detect glycemic drops or spikes—prompting health changes such as increased snacking—when in reality, these values are biologically insignificant.

One major limitation of our pilot study is that it occurred during the COVID-19 pandemic, which restricted recruitment. Our preliminary findings need to be further explored in a larger cohort, and we cannot eliminate the possibility that with a greater sample there would not be a subtle difference in glycemic variability and other measured parameters. However, despite the small sample size, there remained a difference in fasting insulin and HOMA-IR between the two groups, suggesting rising insulin levels being a compensatory process rather than merely the result of a high glycemic load. This was further demonstrated through linear regression analysis, which showed HOMA-IR to be associated with BMI, WC, HbA1c, and fasting insulin levels, while MAGE was not found to be associated with these factors.

Additionally, the best measures of glycemic variability and insulin response remain unknown. MAGE, as extracted from CGM, is viewed as the most comprehensive index for assessment of intraday glycemic variability, but it does not account for insulin responsiveness and other processes of post-prandial cellular metabolism. Future studies should recruit a larger cohort of normoglycemic adults to assess the utility of CGM in predicting dysglycemia and aiding weight loss efforts.

## Conclusion

While there is much excitement surrounding the use of commercial CGM products in management of obesity, our preliminary results suggest that fasting insulin and HOMA-IR values may be more clinically useful than CGM data alone. The absence of increased glycemic variability in normoglycemic individuals is suggestive that the Energy Balance model may represent a more accurate conceptual framework for obesity. Finally, the application of CGM in weight loss should await further trials.

## Data availability statement

The original contributions presented in this study are included in this article, further inquiries can be directed to the corresponding author.

## Ethics statement

The studies involving human participants were reviewed and approved by Northwell Health Institutional Review Board (no. 19-1069). The patients/participants provided their written informed consent to participate in this study.

## Author contributions

DC and MR contributed in conception, design, statistical analysis, and supervised the study. DC, SZ, BF, and AB contributed to data collection, data analysis, and manuscript drafting. All authors had final approval of the submitted and published versions of the manuscript.
